# Supplementary material for: Non-Responsive and Refractory Coeliac Disease: Experience from the NHS England National Centre
Source: Nutrients. 2022 Jul 5;14(13):2776. doi: 10.3390/nu14132776 (PMC9268848; doi:10.3390/nu14132776)
Supplement: Supplementary file 1 [file nutrients-14-02776-s001.zip › nutrients-1761492-supplementary.pdf]

Table S1. Summary of the diagnostic criteria for different pathologies. SBBO = small bowel bacterial overgrowth; IBS = irritable bowel syndrome; IBD = inflammatory bowel disease; RCD = refractory coeliac disease; EATL = enteropathy associated T cell lymphoma

**Disease category**	**Diagnostic criteria**
Dietary gluten exposure	Review by specialist dietitian
Supersensitive	Review by specialist dietitian and symptomatic improvement on a wheat and gluten contamination elimination diet
Gastro-oesophageal reflux disease	Upper GI endoscopy plus/ minus pH monitoring
Reflux dysmotility	Oesophageal manometry
H pylori	CLO test or gastric biopsies
SBBO	Glucose hydrogen breath tests
Lactose/ fructose intolerance	Breath tests
Pancreatic exocrine insufficiency	Faecal elastase testing
Bile acid diarrhoea	SeHCAT scan
IBS	Symptoms which met the Rome criteria for IBS and in whom other causes had been ruled out
Microscopic colitis	Relevant symptoms and histological assessment of colonic biopsies
IBD	Relevant symptoms and histological assessment of colonic biopsies
RCD1	Typical clinical manifestations of CD plus evidence of Marsh 3 lesions on repeat duodenal biopsy, that have persisted, or recurred, despite strict adherence to a GFD (as assessed by a specialist dietitian) for at least 12 months. The IEL phenotype within the duodenal tissue is within normal limits. Other causes of villous atrophy have been excluded.[4,10]
RCD2	As in RCD1, without the absolute requirement for 12 months on a GFD, but with the presence of ≥40% aberrant IEL phenotype on IHC assessment, or ≥20% aberrant IEL phenotype on flow cytometry, and/ or the presence of a detectable clonal TCR rearrangement, in duodenal biopsy specimens.[10,11,19]
Secondary EATL	WHO Classification of Tumours of Haematopoietic and Lymphoid Tissues. The patient is already known to have RCD2.[20]
De novo EATL	WHO Classification of Tumours of Haematopoietic and Lymphoid Tissues. History of typical CD and/ or evidence of Marsh 3 lesions in non-involved mucosa;[20] no prior history, or evidence, of RCD2.
Other GI malignancies	WHO Classification of Tumours of Haematopoietic and Lymphoid Tissues.

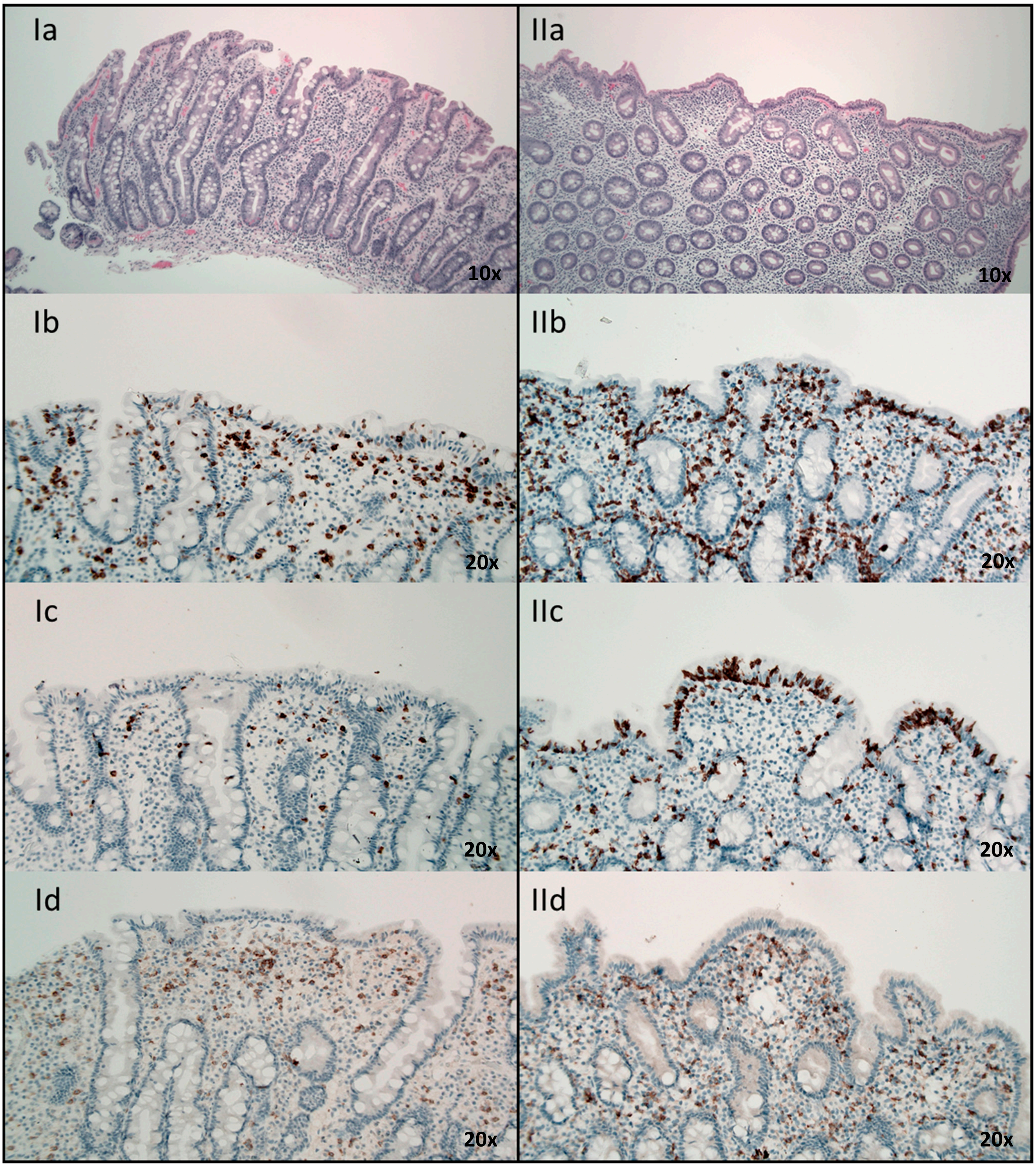

Figure S1. Example of immunohistochemical assessment of duodenal biopsy sections from a patient with RCD2 (Ia-d; left) and typical active CD (IIa-d; right). Ia: Haematoxylin and eosin staining in RCD2 showing complete villous atrophy and crypt hyperplasia; Ib: CD3 staining in RCD2 showing increased intra-epithelial lymphocytes (IELs); Ic: CD8 staining in RCD2 showing that virtually no IELs mark with CD8; Id: CD4 staining in RCD2 confirming absence of T helper cells in the epithelium. IIa: Haematoxylin and eosin staining in typical CD showing complete villous atrophy and crypt hyperplasia; IIb: CD3 staining in typical active CD showing increased IELs; IIc: CD8 staining in typical CD showing that IELs that mark with CD3 also mark with CD8; IId: CD4 staining in typical CD confirming absence of T helper cells in the epithelium.

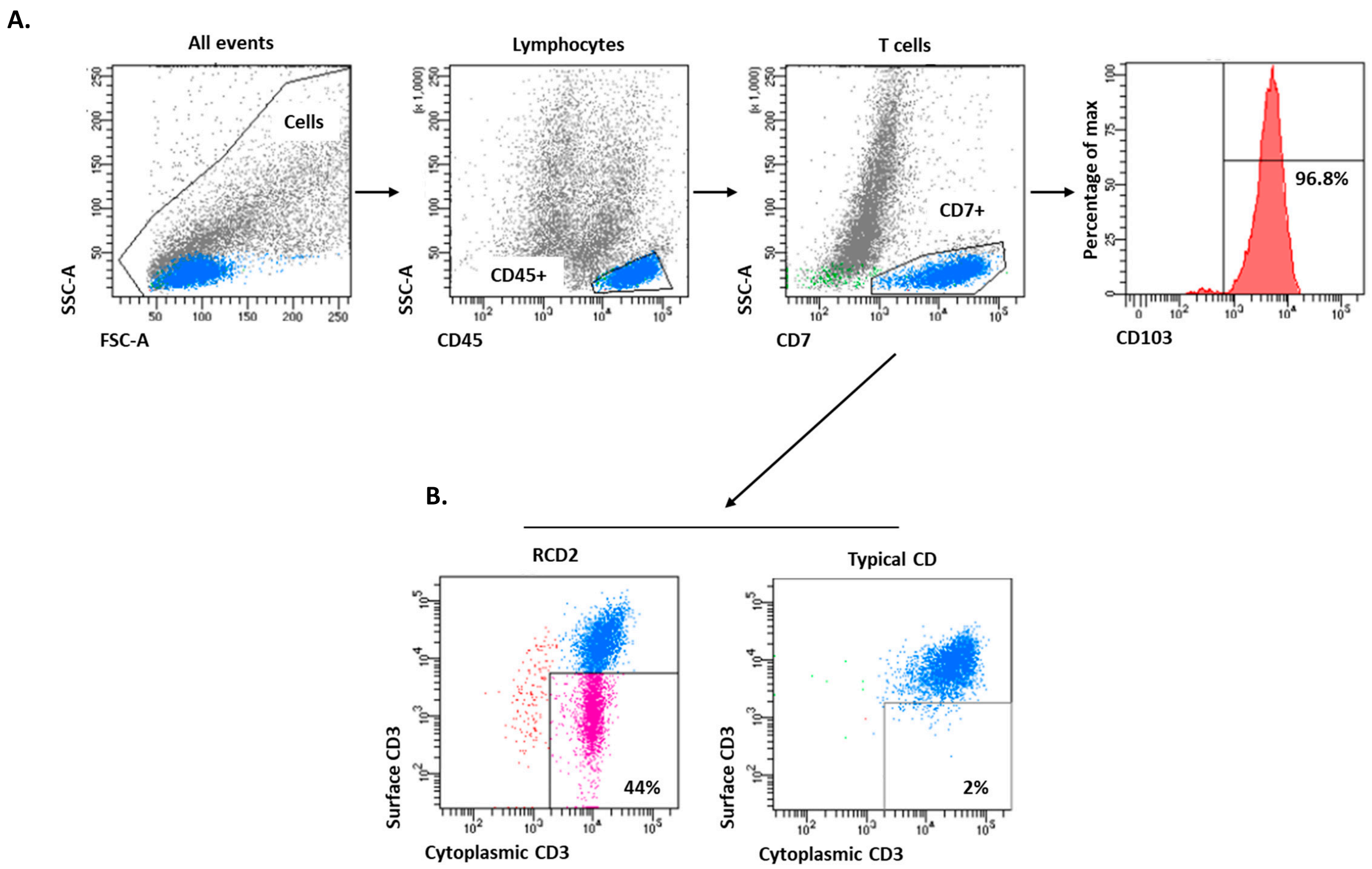

Figure S2. Example flow cytometry gating strategy for identifying the frequency of the aberrant intraepithelial lymphocyte (IEL) population (surface (s)CD3- cytoplasmic (c)CD3+) isolated from duodenal biopsy specimens [12]. A. Lymphocytes were identified as CD45+ with low sideward scatter. Almost all CD45+CD7+ T cells were positive for CD103, which is known to be highly expressed by IELs (19). B. The percentage of sCD3-cCD3+ lymphocytes were enumerated in CD45+CD7+ cells. Representative plots with frequencies of the aberrant phenotype in RCD2 (left) and typical active CD (right).
